# Delta weight loss unlike genetic variation associates with hyperoxaluria after malabsorptive bariatric surgery

**DOI:** 10.1038/s41598-023-35941-8

**Published:** 2023-06-03

**Authors:** Lotte Scherer, Ria Schönauer, Melanie Nemitz-Kliemchen, Tobias Hagemann, Elena Hantmann, Jonathan de Fallois, Friederike Petzold, Matthias Blüher, Jan Halbritter

**Affiliations:** 1 Division of Nephrology, University of Leipzig Medical Center, Leipzig, Germany; 2 Department of Nephrology and Internal Intensive Care Medicine, Charité University, Berlin, Germany; 3 Helmholtz Institute for Metabolic, Obesity and Vascular Research (HI-MAG) of the Helmholtz Zentrum München at the University of Leipzig, Ph.-Rosenthal-Str. 27, 04103 Leipzig, Germany

**Keywords:** Genetics, Endocrinology, Gastroenterology, Nephrology, Urology

## Abstract

The risk of enteric hyperoxaluria is significantly increased after malabsorptive bariatric surgery (MBS). However, its underlying determinants are only poorly characterized. In this case–control study, we aimed at identifying clinical and genetic factors to dissect their individual contributions to the development of post-surgical hyperoxaluria. We determined the prevalence of hyperoxaluria and nephrolithiasis after MBS by 24-h urine samples and clinical questionnaires at our obesity center. Both hyperoxaluric and non-hyperoxaluric patients were screened for sequence variations in known and candidate genes implicated in hyperoxaluria (*AGXT*, *GRHPR*, *HOGA1, SLC26A1*, *SLC26A6*, *SLC26A7*) by targeted next generation sequencing (tNGS). The cohort comprised 67 patients, 49 females (73%) and 18 males (27%). While hyperoxaluria was found in 29 patients (43%), only one patient reported postprocedural nephrolithiasis within 41 months of follow-up. Upon tNGS, we did not find a difference regarding the burden of (rare) variants between hyperoxaluric and non-hyperoxaluric patients. However, patients with hyperoxaluria showed significantly greater weight loss accompanied by markers of intestinal malabsorption compared to non-hyperoxaluric controls. While enteric hyperoxaluria is very common after MBS, genetic variation of known hyperoxaluria genes contributes little to its pathogenesis. In contrast, the degree of postsurgical weight loss and levels of malabsorption parameters may allow for predicting the risk of enteric hyperoxaluria and consecutive kidney stone formation.

## Introduction

Bariatric surgery has substantially reduced morbidity and mortality in severely obese patients^1,2^. Particularly, procedures that induce weight loss through malabsorption, such as Roux-en-Y gastric bypass (RYGB), have become standard of care for patients with a body mass index (BMI) > 40 kg/m^2^^3^. Among the long-term complications of malabsorptive bariatric surgery (MBS), however, is the risk of postoperative enteric hyperoxaluria and the development of calcium oxalate nephrolithiasis (CaOx-NL)^4,5^. Strikingly, patients after RYGB appear to have hyperoxaluria-based nephrolithiasis twice as often as patients after sleeve gastrectomy, a restrictive type of bariatric surgery^6^.

In contrast to enteric hyperoxaluria, primary hyperoxaluria (PH) is an autosomal-recessive condition, resulting from biallelic pathogenic variants in three known disease genes, namely *AGXT* (MIM# 259900), *GRHPR* (MIM# 260000), *HOGA1* (MIM# 613616).

In this study, we aimed at identifying clinical and genetic factors that put patients at risk of developing hyperoxaluria (and consecutive CaOx-NL) after MBS. Therefore, we determined the prevalence of hyperoxaluria in a cohort that underwent MBS by measuring 24-h urinary oxalate excretion. In a next step, hyperoxaluric and non-hyperoxaluric patients were screened for sequence variations in genes known to cause monogenic hyperoxaluria (*AGXT*, *GRHPR*, *HOGA1; SLC26A1*—MIM# 167030) and candidate genes involved in renal and intestinal oxalate homeostasis (*SLC26A6*, *SLC26A7*) by targeted next generation sequencing (tNGS). Furthermore, we analyzed clinical parameters, such as kidney function, postsurgical weight loss and serum levels of malabsorption markers to evaluate potential differences between hyperoxaluric and non-hyperoxaluric patients.

## Methods

### Study participants

Study participants were recruited between October 2018 and February 2020 from the IFB Obesity Outpatient Clinic for Adults at the University of Leipzig Medical Center (Germany). Inclusion criteria for enrollment were age > 18 years and a history of MBS (RYGB) at our center. Patients who underwent restrictive bariatric surgery were excluded. Indications for bariatric surgery included a BMI ≥ 40 kg/m^2^ or a BMI ≥ 35 kg/m^2^ with one or more of the following: type 2 diabetes, coronary artery disease, hypertension, heart failure, nephropathy, obstructive sleep apnea syndrome, obesity-hypoventilation syndrome, gastroesophageal reflux disease, nonalcoholic fatty liver disease (NAFLD) or nonalcoholic steatohepatitis, idiopathic intracranial hypertension, bronchial asthma, chronic venous insufficiency, urinary incontinence, immobilizing joint disease, reduced fertility or polycystic ovary syndrome.

### Data collection

At the day of recruitment, patients were requested to provide 24-h urine specimen that were analyzed for oxalate, creatinine, and urine volume. Urinary oxalate levels/day were calculated by multiplying the oxalate concentration by urinary volume. Patients’ pre-op values of kidney function (creatinine, eGFR) were obtained through electronic health records. Blood parameters included serum creatinine, eGFR (Chronic Kidney Disease Epidemiology Collaboration (CKD-EPI) formula), urea, uric acid, calcium, triglycerides, HDL-C, LDL-C, HbA1c, total protein, albumin, zinc, and iron. The medical record of each patient was abstracted for the following information: current age, pre-/post procedure weight and BMI (pre-procedure = latest value before surgery; post procedure = day of recruitment), age at time of operation, details of bariatric procedure, interval between procedure and urine analysis, history of pre-/post-procedural kidney stones and secondary diseases like type 2 diabetes, arterial hypertension and NAFLD. To determine the weight loss parameter ‘Excess weight loss (EWL)’, the following calculation was performed: $\left( {\frac{{{\text{Preop. Weight}} - {\text{Follow-Up Weight}}\# \# }}{{{\text{Preop. Weight}} - {\text{Ideal Body Weight}}\# }}} \right) \times 100$. (^#^for ideal body weight we used the Metropolitan Life Insurance Company (MetLife) Height and Weight tables^7^ and chose the maximum weight on the charts; ^##^follow-up weight was the patients’ weight at recruitment).

### Gene panel-analysis

The gene panel covered exon–intron boundaries and coding regions of *AGXT*, *GRHPR*, *HOGA1*, *SLC26A1*, *SLC26A6*, and *SLC26A7*. Library preparation for tNGS consisted of amplification of pre-defined genetic regions using microfluidic multiplex PCR, barcoding of distinctive patient sequences and purification of the sample pool^8^. High-throughput sequencing was performed using the *MiSeq® System* (Illumina, USA).

*SEQUENCE Pilot, module SeqNext v4.3.1* by JSI medical systems (Germany) was used for variant calling. Sequencing data was queried for genomes and exomes within the *Genome Aggregation Database* (gnomAD) and entries in *ClinVar* (https://www.ncbi.nlm.nih.gov/clinvar/)^9^ using *Query Tabular*^10^ on the Galaxy Server (http://galaxy.bioinf.uni-leipzig.de/). Additionally, the variant tables were annotated on the *Combined Annotation Dependent Depletion* (CADD)^11^ website including scaled CADD score. Variant tables were filtered by region of interest (exons and splice site variants ranging from − 12 to + 8 bp), non-synonymous consequence, gnomAD allele frequency (AD: ≤ 0.1%, AR: ≤ 1%), inheritance (AR: homozygous or compound heterozygous), in silico prediction (SIFT, PolyPhen, ClinVar, and scaled CADD score ≥ 10). Variant classification was performed in agreement with the published diagnostic criteria of the American College of Medical genetics and Genomics (ACMG)^12^: class I to class V—with class IV–V defined as diagnostic.

### Statistics

All statistical analysis were performed using Graphpad PRISM 9.1.0. (GraphPad Software Inc., La Jolla, CA, USA). Descriptive statistics were expressed as mean ± S.E.M. Student’s *t*-test and one-way ANOVA were used to analyze differences in means for normally distributed data. Means between groups in non-normally distributed data were analyzed by Mann–Whitney and Kruskal–Wallis test. Differences in means between pre- and postprocedural values within normally-distributed groups were evaluated by paired Student’s *t*-test and in groups with non-normal distribution Wilcoxon test was used. Fisher’s Exact test was used to compare categorical variables. Statistical significance was defined as p < 0.05.

### Ethical approval

The Leipzig Obesity BioBank (LOBB, https://www.helmholtz-munich.de/en/hi-mag/cohort/leipzig-obesity-bio-bank-lobb) was approved by the Ethics committee of the University of Leipzig (Ethics vote 017-12-23012012). The study participants gave their written informed consent prior to tissue sampling and data collection. For patients not included in the LOBB, written informed consent was obtained before blood collection (Ethics vote 159/14-ff. University of Leipzig). All methods were performed in accordance with the relevant guidelines and regulations. All raw data used for statistical analysis is available in the Supplement.

## Results

### Overall characteristics of the study cohort

**Figure 1:**
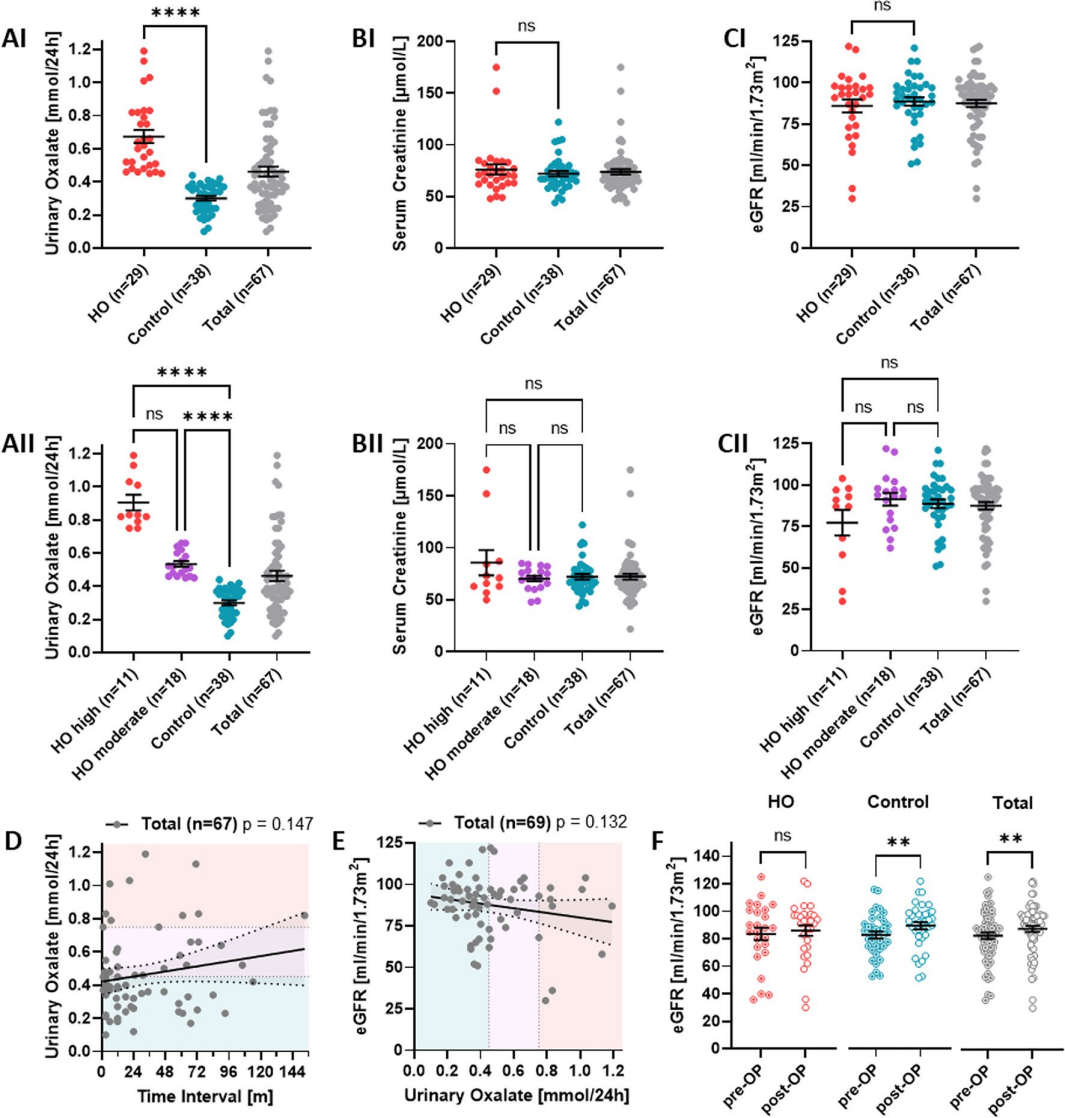
Postsurgical hyperoxaluria is associated with reduced kidney function. (**AI**–**CI**) Scatter plots showing mean ± SEM values of urinary oxalate excretion, serum creatinine and eGFR in HO, control and total. Mann–Whitney test was used for statistical analysis. (**AII**–**CII**) Scatter plots showing mean ± SEM values of urinary excretion, serum creatinine and eGFR in ‘HO-high’, ‘HO-moderate’, control, and total. Ordinary one-way ANOVA and Kruskal–Wallis test was used for statistical analysis. (**D**) Simple linear regression of urinary oxalate excretion and follow-up time in months (total cohort). (**E**) Simple linear regression of eGFR and urinary oxalate excretion (total cohort). (**F**) Scatter plot showing pre- and postoperative mean ± SEM eGFR levels in HO, control and total. Wilcoxon matched-pairs signed rank test was used for statistical analyses. ****p < 0.0001; ***p < 0.001; **p < 0.01; *p < 0.05; *eGFR* estimated glomerular filtration rate (CKD-EPI), *HO* hyperoxaluria, *ns* not significant.

The study cohort (Table 1) consisted of 67 adult patients, 49 females (73%) and 18 males (27%), with previous MBS. Medical records revealed a pre-procedural BMI ≥ 40 kg/m^2^ in most patients (87%), a BMI ≥ 35 kg/m^2^ in 13% and < 35 kg/m^2^ in 1% resulting in a mean BMI before surgery of 45.7 ± 0.8 kg/m^2^ for the overall cohort. MBS procedures comprised RYGB. RYGB details showed a mean length of 58.4 ± 1.7 cm in the biliopancreatic limb and 152.4 ± 0.8 cm in the alimentary (roux) limb. Fourteen patients were converted from previous bariatric procedures including sleeve gastrectomy (n = 7), gastric banding (n = 3) and gastric balloon (n = 2). The mean age at the time of surgery was 51.9 years (range 24–70 years) and the mean time interval between bariatric surgery and follow-up time was 41.6 ± 4.0 months. Hyperoxaluria was defined as urinary oxalate excretion ≥ 0.45 mmol/day. To investigate the differences between hyperoxaluric and non-hyperoxaluric patients we divided the cohort primarily into the two main groups ‘hyperoxaluria (HO)’ (urinary oxalate excretion ≥ 0.45 mmol/day) and controls (< 0.45 mmol/day). Accordingly, an increased level of urinary oxalate excretion was detected in 29 out of 67 individuals (43%, 23 women and 7 men) with a mean age of 54.0 years (range 27–72 years). Their mean urinary oxalate excretion was 0.67 ± 0.04 mmol/day, thus being significantly higher than in controls (0.30 ± 0.01; p < 0.0001) (Table 1; Fig. 1AI). In addition, we further divided the hyperoxaluric patients into two subgroups to determine differences between patients with moderate (‘HO moderate’; urinary oxalate excretion ≥ 0.45–0.74 mmol/day) and high levels (‘HO high’; urinary oxalate excretion ≥ 0.75 mmol/day) of oxalate excretion (Table 1; Fig. 1AII). Urinary oxalate levels were significantly different between all subgroups (‘HO high’ versus control: p < 0.0001 and ‘HO moderate’ versus control: p < 0.0001) except for ‘HO high’ versus ‘HO moderate’ with p = 0.16 (Fig. 1AII). For urinary albumin, values were significantly lower in hyperoxaluric patients than in controls (12.5 ± 2.9 versus 33.2 ± 7.7) (p = 0.009) (Table 2).

**Table 1:** Clinical and metabolic differences between hyperoxaluric and non-hyperoxaluric patients.

Parameters	Hyperoxaluria (n = 29) ≥ 0.45 mmol/day	HO high (n = 11) ≥ 0.75 mmol/d	HO moderate (n = 18) 0.45–0.74 mmol/d	Control (n = 38) < 0.45 mmol/day	Total (n = 67)	HO vs control	HO high vs HO moderate	HO high vs control	HO moderate vs control
Age at follow-up (years**)**	54.0 ± 2.2 (n = 29)	56 ± 2.4 (n = 11)	52.8 ± 3.2 (n = 18)	55.1 ± 1.8 (n = 38)	54.6 ± 1.4 (n = 67)	0.69	0.73	0.97	0.75
Sex									
Male, n (%)	n = 7/29 (24%)	n = 1/11 (9%)	n = 6/18 (33%)	n = 11/38 (29%)	n = 18/67 (27%)	> 0.9999	nd	nd	nd
Female, n (%)	n = 22/29 (76%)	n = 10/11 (91%)	n = 12/18 (67%)	n = 27/38 (71%)	n = 49/67 (73%)				
Follow-up-time (months)	46.6 ± 6.8 (n = 29)	53.5 ± 13.3 (n = 11)	42.4 ± 7.5 (n = 18)	37.7 ± 4.7 (n = 38)	41.6 ± 4.0 (n = 67)	0.52	> 0.99	> 0.99	> 0.99
Urine									
Oxalate/24 h (mmol/day)	0.67 ± 0.04 (n = 29)	0.91 ± 0.05 (n = 11)	0.53 ± 0.02 (n = 18)	0.30 ± 0.01 (n = 38)	0.46 ± 0.03 (n = 67)	< 0.0001 (****)	0.16	< 0.0001 (****)	< 0.0001 (****)
Blood									
Creatinine (µmol/L)	76.1 ± 5.0 (n = 29)	85.6 ± 12.2 (n = 11)	70.4 ± 2.6 (n = 18)	72.1 ± 2.7 (n = 38)	73.9 ± 2.6 (n = 67)	0.64	> 0.99	> 0.99	> 0.99
eGFR pre-OP (mL/min/1.73 m^2^)	83.6 ± 4.6 (n = 25)	74.7 ± 9.6 (n = 9)	88.5 ± 4.6 (n = 16)	82.0 ± 2.6 (n = 36)	82.6 ± 2.4 (n = 61)	0.74	0.19	0.56	0.47
eGFR post-OP (mL/min/1.73 m^2^)	86.0 ± 3.9 (n = 29)	77.2 ± 7.7 (n = 11)	91.4 ± 3.8 (n = 18)	88.7 ± 2.6 (n = 38)	87.5 ± 2.2 (n = 67)	0.89	0.54	0.90	> 0.99
eGFR-delta (mL/min/1.73 m^2^)	1.5 ± 2.8 (n = 25)	-0.18 ± 4.9 (n = 9)	2.4 ± 3.4 (n = 16)	6.3 ± 1.8 (n = 36)	4.3 ± 1.6 (n = 61)	0.19	> 0.99	0.51	> 0.99
Total protein (g/L)	68.6 ± 0.7 (n = 28)	68.1 ± 1.0 (n = 11)	69.0 ± 0.9 (n = 17)	70.9 ± 0.7 (n = 37)	70.0 ± 0.5 (n = 65)	0.02 (*)	0.81	0.09	0.22
Zinc (µmol/L)	10.4 ± 0.4 (n = 28)	9.5 ± 0.2 (n = 11)	11.1 ± 0.7 (n = 17)	10.9 ± 0.4 (n = 35)	10.7 ± 0.3 (n = 63)	0.11	0.31	0.07	> 0.99
Weight loss									
BMI pre-OP (kg/m^2^)	46.1 ± 1.0 (n = 29)	46.7 ± 1.6 (n = 11)	45.8 ± 1.4 (n = 18)	44.5 ± 1.0 (n = 38)	45.2 ± 0.7 (n = 67)	0.18	> 0.99	0.66	0.99
BMI post-OP (kg/m^2^)	33.4 ± 1.0 (n = 29)	32.7 ± 2.0 (n = 11)	33.8 ± 1.2 (n = 18)	35.3 ± 1.0 (n = 38)	34.5 ± 0.7 (n = 67)	0.2	0.64	0.51	0.63
BMI-delta (kg/m^2^)	12.7 ± 1.2 (n = 29)	14.0 ± 2.2 (n = 11)	11.9 ± 1.4 (n = 18)	9.2 ± 0.9 (n = 38)	10.7 ± 0.8 (n = 67)	0.02 (*)	0.65	0.06	0.24
Weight-delta (kg)	35.5 ± 3.1 (n = 29)	37.7 ± 5.2 (n = 11)	34.0 ± 4.0 (n = 18)	26.3 ± 2.7 (n = 38)	30.3 ± 2.1 (n = 67)	0.02 (*)	> 0.99	0.14	0.27
Weight loss (%)	27.0 ± 2.3 (n = 29)	29.6 ± 4.1 (n = 11)	25.5 ± 2.8 (n = 18)	20.4 ± 1.8 (n = 38)	23.3 ± 1.5 (n = 67)	0.03 (*)	0.37	0.03 (*)	0.14
EWL max. frame (%)	62.5 ± 5.4 (n = 29)	67.2 ± 8.9 (n = 11)	59.6 ± 6.9 (n = 18)	50.6 ± 4.9 (n = 38)	55.8 ± 3.7 (n = 67)	0.11	0.79	0.25	0.55

*BMI* body mass index, *eGFR* estimated glomerular filtration rate, *EWL* excess body weight; ****p < 0.0001; *p < 0.05.

**Table 2:** Differences in serum chemistries and urinalysis between hyperoxaluric and non-hyperoxaluric patients.

Parameters	Hyperoxaluria (n = 29) ≥ 0.45 mmol/day	HO high (n = 11) ≥ 0.75 mmol/day	HO moderate (n = 18) 0.45–0.74 mmol/day	Control (n = 38)	Total (n = 67)	HO vs control	HO high vs HO moderate	HO high vs control	HO moderate vs control
Urine									
Volume (mL/day)	1844.5 ± 114.6 (n = 29)	1536.4 ± 128.8 (n = 11)	2032.8 ± 152.9 (n = 18)	1802.2 ± 132.5 (n = 38)	1820.5 ± 89.4 (n = 67)	0.77	0.13	0.72	0.60
Oxalate (mmol/L)	0.42 ± 0.05 (n = 26)	0.59 ± 0.09 (n = 9)	0.31 ± 0.04 (n = 17)	0.22 ± 0.02 (n = 37)	0.30 ± 0.03 (n = 63)	< 0.0001 (****)	0.048 (**)	< 0.0001 (****)	0.08
Albumin (mg/L)	12.5 ± 2.9 (n = 19)	11.8 ± 2.6 (n = 9)	13.1 ± 5.2 (n = 10)	33.2 ± 7.7 (n = 23)	23.8 ± 4.7 (n = 42)	0.009 (**)	> 0.99	0.22	0.06
Blood									
Urea (mmol/L)	5.5 ± 0.4 (n = 28)	6.0 ± 0.9 (n = 11)	5.2 ± 0.4 (n = 17)	5.4 ± 0.3 (n = 37)	5.5 ± 0.3 (n = 65)	0.70	> 0.99	> 0.99	> 0.99
Uric acid (µmol/L)	293.5 ± 13.4 (n = 28)	261.6 ± 26.0 (n = 11)	314.2 ± 12.5 (n = 17)	317.4 ± 16.1 (n = 36)	307.0 ± 10.8 (n = 64)	0.51	0.07	0.13	> 0.99
Triglycerides (mmol/L)	1.3 ± 0.1 (n = 28)	1.4 ± 0.2 (n = 11)	1.3 ± 0.2 (n = 17)	1.4 ± 0.1 (n = 36)	1.4 ± 0.1 (n = 64)	0.56	> 0.99	> 0.99	> 0.99
LDL-C (mmol/L)	2.4 ± 0.2 (n = 28)	2.5 ± 0.3 (n = 11)	2.3 ± 0.1 (n = 17)	2.4 ± 0.1 (n = 36)	2.4 ± 0.1 (n = 64)	0.51	> 0.99	> 0.99	> 0.99
HDL-C (mmol/L)	1.4 ± 0.1 (n = 28)	1.4 ± 0.2 (n = 11)	1.4 ± 0.1 (n = 17)	1.5 ± 0.1 (n = 36)	1.4 ± 0.1 (n = 64)	0.70	> 0.99	> 0.99	> 0.99
Calcium (mmol/L)	2.4 ± 0.0 (n = 29)	2.4 ± 0.0 (n = 11)	2.4 ± 0.0 (n = 18)	2.4 ± 0.0 (n = 38)	2.4 ± 0.0 (n = 67)	0.99	0.99	0.99	0.99
HbA1c (%)	5.5 ± 0.1 (n = 28)	5.6 ± 0.3 (n = 11)	5.4 ± 0.1 (n = 17)	5.9 ± 0.2 (n = 36)	5.7 ± 0.1 (n = 64)	0.12	> 0.99	> 0.99	0.42
Albumin (g/L)	43.7 ± 0.5 (n = 28)	43.0 ± 1.0 (n = 11)	44.0 ± 0.6 (n = 17)	44.5 ± 0.4 (n = 38)	44.1 ± 0.3 (n = 66)	0.20	0.68	0.38	0.68
Iron (µmol/L)	16.1 ± 1.5 (n = 28)	13.7 ± 1.5 (n = 11)	17.7 ± 2.2 (n = 17)	15.9 ± 1.0 (n = 35)	16.0 ± 0.9 (n = 63)	0.54	0.33	0.55	0.55

****p < 0.0001; **p < 0.01.

### Postsurgical hyperoxaluria is related to reduced kidney function

Post-surgery kidney function was estimated by serum creatinine levels and eGFR at the individual follow-up time point and showed no significant differences between the patient groups HO and control (Table 1; Fig. 1BI ,CI). When dividing into subgroups, differences in creatinine remained insignificant (Table 1; Fig. 1BII). However, ‘HO high’ patients exhibited a reduced mean eGFR compared to ‘HO moderate’ patients (Table 1; Fig. 1CII). In addition, we observed a trend for higher urinary oxalate excretion values with increasing follow-up time post-surgery, which was, however, characterized by a remarkable variability at any time point (Fig. 1D). In line with the overall lower kidney function in the ‘HO high’ group, we detected a tendency for lower eGFR values in patients with higher oxalate excretion (Fig. 1E). Interestingly, when comparing pre- and postoperative mean eGFR values, we found a significant increase (p = 0.0014) within the control group but not in hyperoxaluric patients (p = 0.36) (Fig. 1F).

### Impact of hyperoxaluria-associated genes in the development of postsurgical hyperoxaluria

**Figure 2:**
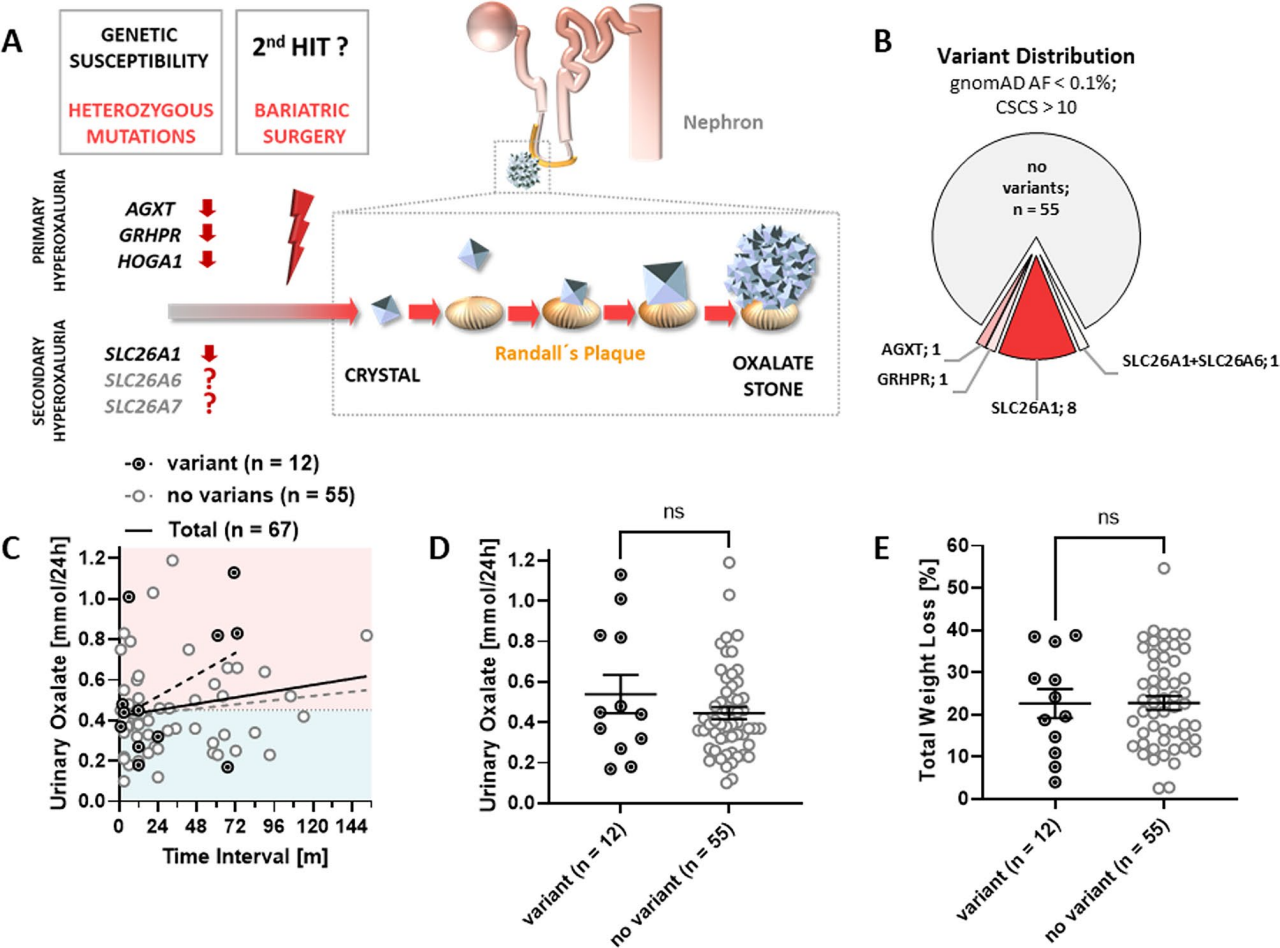
Impact of hyperoxaluria-associated genes in the development of postsurgical hyperoxaluria. (**A**) Hypothetical model of Ca-Ox stone formation after bariatric surgery. Potential stone formers carry a genetic susceptibility in way of predisposing pathogenic heterozygous variants in either PH-genes (AGXT/GRHPR/HOGA1) or in genes encoding enteric/renal oxalate transporters (SLC26A1, SLC26A6, SLC26A7). A second “hit”, however, in form of bariatric surgery and consecutive enteric oxalate over-absorption may be required to increase urinary supersaturation, initiate crystallization, aggregation of crystals at Randall’s plaque, and eventual urinary calculus formation. Adapted from Pfau/Knauf^13^. (**B**) Pie chart indicating genetic findings and variant distribution in total post bariatric cohort. (**C**) Simple linear regression of urinary oxalate excretion and follow-up time in patients with variants, no variants and total. (**D**,**E**) Scatter plots showing mean ± SEM values of urinary oxalate excretion and total weight loss in patients with variants and no variants. Mann–Whitney and student’s unpaired *t*-test was used for statistical analysis. *AF* allele frequency, *CSCS* c-scaled CADD score, *gnomAD* Genome Aggregation Database, *ns* not significant.

To evaluate the contribution of genetic factors in the development of hyperoxaluria after bariatric surgery, we conducted tNGS to detect variations within genes known to cause monogenic hyperoxaluria and Ca-Ox-NL (*AGXT*, *GRHPR*, *HOGA1; SLC26A1*) as well as the candidate genes *SLC26A6* and *SLC26A7* (Fig. 2A). We hypothesized that MBS-associated enteric hyperoxaluria manifests upon genetic susceptibility, which is conveyed via hypomorphic variants in genes involved in hepatic, renal, and intestinal oxalate homeostasis. The mean coverage of tNGS was 477× for analyzed regions of interest. However, diagnostic validity was limited by an amplicon drop-out rate of 9%. Within the six genes analyzed, we identified 13 variants in 12 patients, 6 of which were hyperoxaluric (HO) and 6 non-hyperoxaluric (control) (HO: 1 × *GRHPR*, 5 × *SLC26A1*; control: 1 × *AGXT*, 3 × *SLC26A1*, 1 × *SLC26A7*, 1 × *SLC26A1* + *SLC26A6*) (Fig. 2B; Table S1). By analyzing individual oxalate excretion values over follow-up time, we found a trend for higher urinary oxalate excretion with increasing time in patients with genetic findings versus patients without findings (Fig. 2C). However, this result was of limited significance regarding the high variability at each time point and the low number of values at longer time intervals. In addition, the mean urinary oxalate level in variant carriers was not significantly increased in comparison to non-variant carriers (0.539 ± 0.09 mmol/day and 0.445 ± 0.030 mmol/day, respectively; p = 0.55) (Fig. 2D). Weight loss between both groups was slightly lower in patients with genetic findings (22.6 ± 3.5% versus 22.7 ± 1.6%; p = 0.97) (Fig. 2E). In summary, we found no evidence for an impact of variation in known or candidate hyperoxaluria-associated genes in the development of postsurgical hyperoxaluria in our cohort.

### Association of hyperoxaluria with weight loss and enteric malabsorption

**Figure 3:**
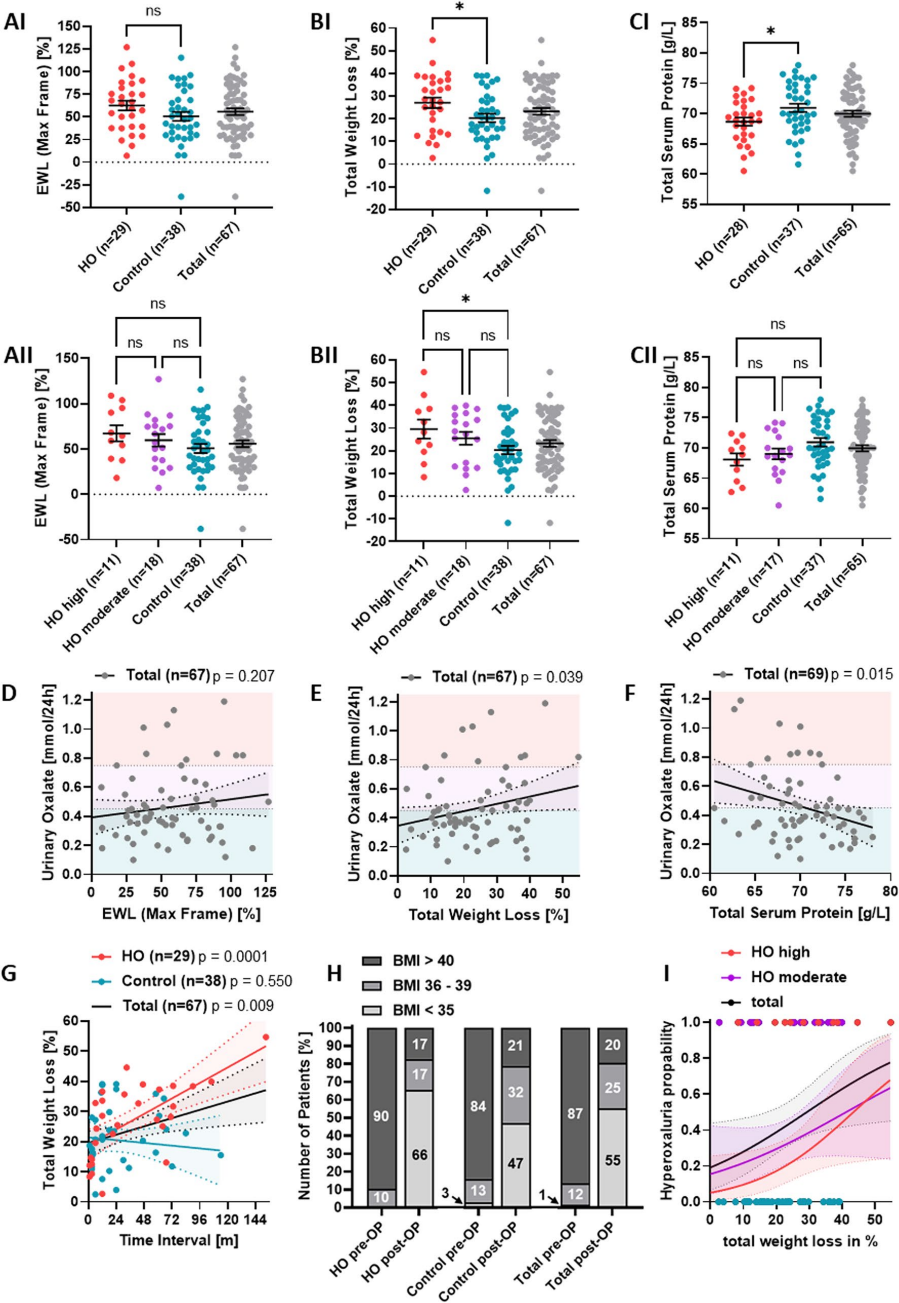
Association of hyperoxaluria with weight loss and enteric malabsorption. (**AI**–**CI**) Scatter plots showing mean ± SEM values of EWL, total weight loss and total serum protein in HO, control and total. Student’s unpaired *t*-test was used for statistical analysis. (**AII**–**CII**) Scatter plots showing mean ± SEM values of EWL, total weight loss and total serum protein in ‘HO-high’, ‘HO-moderate’, control and total. Ordinary one-way ANOVA was used for statistical analysis. (**D**–**F**) Simple linear regression of urinary oxalate excretion with EWL, total weight loss and total serum protein, showing significant correlation for higher oxalate excretion with higher total weight loss and lower total serum protein (total cohort). (**G**) Simple linear regression of total weight loss and follow-up time showing significant correlation between weight loss and follow-up time for HO and total. (**H**) Spectrum of BMI-groups pre- and postoperatively between HO, control and total. (**I**) Simple logistic regression showing the likelihood of developing hyperoxaluria with weight loss as a predictor variable. Log-likelihood ratio test (LRT) was used for statistical analysis. *p < 0.05; *BMI* body mass index, *EWL* excess weight loss, *HO* hyperoxaluria, *ns* not significant.

We compared different weight loss parameters between HO and controls (Table 1). Excess weight loss (EWL; percentage of overweight that has been lost after surgery) was higher in hyperoxaluric patients compared to controls (HO: 62.5 ± 5.4%; control: 50.6 ± 4.9%) (p = 0.11) (Table 1; Fig. 3AI). In line with these observations, EWL was highest in patients with severe hyperoxaluria (‘HO high’) compared to moderate hyperoxaluria (‘HO moderate’) and controls (Table 1; Fig. 3AII); however, differences between groups remained non-significant (‘HO high’: 67.2 ± 8.9%; ‘HO moderate’: 59.6 ± 6.9%). In contrast, for total weight loss, hyperoxaluric patients showed significantly higher weight reduction when compared with controls (HO: 27.0 ± 2.3%; control: 20.4 ± 1.8%) (p = 0.03) (Table 1; Fig. 3BI), a phenomenon that was also observed for the HO subgroups (‘HO high’: 29.6 ± 4.1%; ‘HO moderate’: 25.5 ± 2.8%) with weight loss in ‘HO high’ being significantly higher than in controls (p = 0.03) (Table 1; Fig. 3BII). To evaluate potential differences in the degree of enteric absorption we analyzed serum levels of total protein and zinc (Table 1) as well as albumin and iron (Table 2). Indeed, hyperoxaluric patients exhibit significantly lower total serum protein levels compared to controls (HO: 68.6 ± 0.7 g/L; control: 70.9 ± 0.7 g/L) (p = 0.02), indicating a higher degree of malabsorption (Table 1; Fig. 3CI), a trend that was also observed by comparing subgroups (Table 1; Fig. 3CII). Levels of zinc (Table 1) and albumin (Table 2) were also lower in HO (p = 0.1 and p = 0.2 respectively). In contrast, comparison of lipid metabolism (LDL-C, HDL-C, and triglycerides) and serum calcium presented non-significant in-between groups (Table 2).

In addition to grouped analyses, individual oxalate levels were analyzed to test for correlations with EWL, total weight loss, and total serum protein. In contrast to EWL, for which only a positive trend could be observed (Fig. 3D), total weight loss significantly correlated with urinary oxalate levels in the total cohort (r = − 0.3; p = 0.02) (Fig. 3E). Accordingly, we observed a significant negative correlation between total serum protein and urinary oxalate levels for all patients (r = 0.30; p = 0.01) (Fig. 3F). Furthermore, a significant positive correlation was detected between weight loss and follow-up time for hyperoxaluric patients (r = 0.66; p = 0.0001), whereas no relationship could be observed for non-hyperoxaluric patients (r = − 1.00, p = 0.55) (Fig. 3G). To illustrate changes in weight loss pre- and postoperatively between HO and control patients, we analyzed the percentage of patients belonging to different BMI-groups (BMI: < 35; 36–39; > 40 kg/m^2^) (Fig. 3H). Compared to controls, the HO group preoperatively included a higher percentage of patients within the highest BMI group (BMI > 40 kg/m^2^: 90% vs 84%) and also a higher percentage within the lowest BMI group postoperatively (BMI: < 35 kg/m^2^: 66% vs 47%). Logistic regression aimed to assess the likelihood of developing hyperoxaluria with weight loss as a predictor variable. Log-likelihood ratio test revealed statistical significance for `HO-high` and ‘total’ (Fig. 3I) (p = 0.0251 and p = 0.0228, respectively). Altogether, our analyses indicate a strong connection between the degree of body weight reduction and enteric hyperoxaluria.

### Association of hyperoxaluria and secondary diagnoses

We evaluated patients’ medical records for secondary confounding diagnoses associated with hyperoxaluria and nephrolithiasis, namely arterial hypertension, type 2 diabetes and NAFLD (Table S2). At the time of recruitment, 31% of hyperoxaluric patients suffered from diabetes versus 26% of controls (p = 0.79) (Figure S1AI; Table S2). Urine oxalate levels of diabetic patients were at 0.47 ± 0.04 mmol/day, thus being insignificantly higher compared to non-diabetic patients (0.46 ± 0.04 mmol/day; p = 0.16) (Figure S1AII; Table S2). HbA1c-levels also revealed no significant difference between HO and control (HO: 5.5 ± 0.1%; control: 5.9 ± 0.2; p = 0.12) (Table 2). Regarding arterial hypertension, no differences between the two groups were observed (72% in HO patients and 76% in control; p > 0.78) (Figure S1BI; Table S2). Oxalate levels in patients with hypertension were non-significantly lower (0.45 ± 0.03 mmol/day) compared to patients without this diagnosis (0.50 ± 0.07 mmol/day; p = 0.85) (Figure S1BII; Table S2). Regarding NAFLD, we only had information about whether patients suffered from the condition at the time of bariatric procedure but not at the time of recruitment. Pre-procedural NAFLD was present in 38% of hyperoxaluric patients and in 34% of control (p = 0.48) (Figure S1CI; Table S2) with no difference between oxalate levels between NAFLD and non-NAFLD patients (0.45 ± 0.04 mmol/day and 0.47 ± 0.04 mmol/day, respectively; p > 0.80) (Figure S1CII; Table S2).

## Discussion

Our study shows a high prevelance of hyperoxaluria in a cohort of patients that underwent malabsorptive bariatric surgery: 43% of our patients presented values higher than 0.45 mmol/day over a mean follow-up time of 46.6 ± 6.8. Those results are in line with other studies that measured oxalate excretion after bariatric surgery. Valezi et al.^14^ compared pre- and 1-year postoperative values and reported a significant increase in urinary oxalate excretion after RYGB with hyperoxaluria found in more than half of all patients, compared to only 4% before the intervention. Similarly, in a study by Park et al.^15^ hyperoxaluria increased from 11% preoperatively to 42% after surgery. Numerous studies reported also an increased risk of hyperoxaluric nephrolithiasis in postbariatric patients after malabsorptive procedures^15–20^.

Unexpectedly, only one patient (1.5%) in our cohort exhibited postoperative Calcium-Oxalate-nephrolithiasis. Time between surgical procedure and stone formation was 3.3 years (40 months). The low number of postoperative nephrolithiasis in our cohort may be explained by the relatively short observation period, as in 67% of patients the time between MBS and the last follow-up was less than 40 months. Secondly, the cohort shows a high variability within follow-up times (range 1–153 months). This leads to possibly missed stone events later in the postoperative period. To address this limitation, we extended the follow-up period for stone formation by abstracting medical records for another 12 months, however, no further stone event was recorded.

The observation that bariatric surgery has a positive effect on kidney function (creatinine-based eGFR) is in line with other studies^21,22^. Overall, values of eGFR and creatinine improved significantly (eGFR: p = 0.0013 and creatinine: p = 0.0003, respectively) within our cohort when comparing pre- and postoperative values. In 2016, Chang et al.^22^ compared 985 severely obese patients undergoing bariatric surgery (mainly RYGB) with 985 matched controls with up to 9 years of follow-up. They reported a 58% risk reduction of eGFR decline and a 57% risk reduction in doubling of serum creatinine or end-stage kidney disease (ESKD) compared with matched non-surgery patients. Imam et al.^21^ studied kidney related outcomes of CKD stage 3 and 4 patients after bariatric surgery matched with an obese control group. They reported that eGFR in the bariatric group was 9.84 mL/min/1.73 m^2^ higher than in controls at a median follow-up of 3 years.

Interestingly, improvement in kidney function between pre- and postoperative values (eGFR and creatinine) was only present in our non-hyperoxaluric control group (Fig. 1F). This could be related to the fact that oxalate is a toxic metabolite^23^ and may exert negative effects on kidney and other organ function. In a recent study, higher 24-h urinary oxalate excretion was found to be a risk factor for chronic kidney disease (CKD) progression and ESKD in individuals with CKD stages 2–4^24^. Additionally, plasma oxalate is known to increase with decreasing eGFR^25^. An inverse correlation between plasma oxalate and eGFR was described in PH-patients even at early CKD stages (stages 1–3b)^26^. And lastly, high plasma oxalate levels were found to increase the risk of sudden cardiac death in patients on dialysis^27^.

Regarding factors that put patients at risk for developing of post-surgical hyperoxaluria, we aimed to characterize genetic susceptibility including risk-alleles or hypomorphic variants next to environmental factors. Although predisposing genetic factors could not be defined, genetic susceptibility conveyed through sequence variants in other candidate genes cannot be excluded by our study.

**Figure 4:**
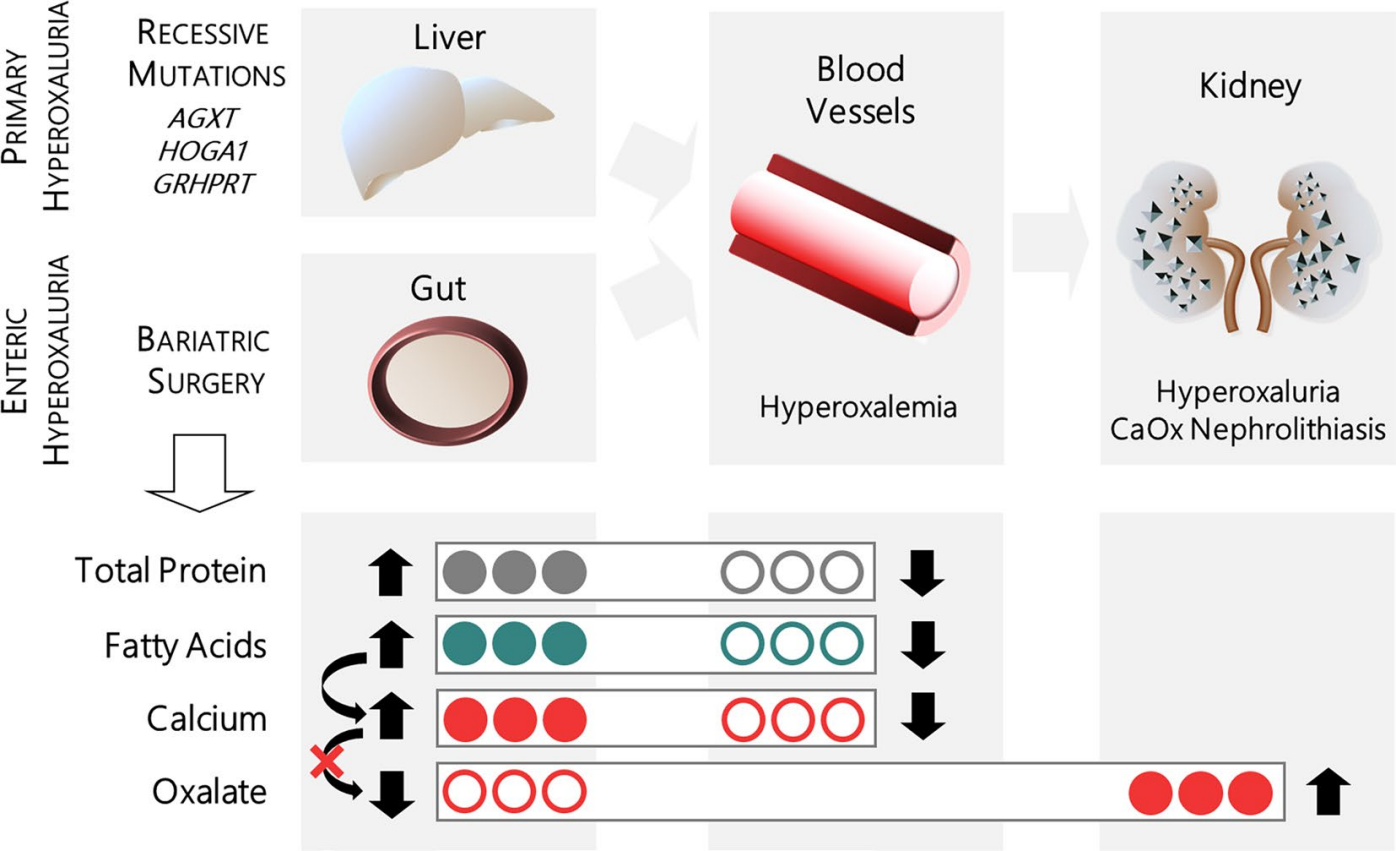
Summary figure. Urinary oxalate derives from two main sources: endogenous oxalate production in the liver as well as absorption of exogenous oxalate in the intestine. In primary hyperoxaluria (PH), an overproduction of oxalate in the liver results in hyperoxalemia and consecutively hyperoxaluria with possible kidney stone formation due to biallelic mutations in the genes AGXT, GRHPR, or HOGA1. These genes encode for peroxisomal or mitochondrial liver enzymes involved in pyruvate/glyoxylate metabolism. Malabsorptive bariatric surgery can cause enteric (secondary) hyperoxaluria. Enteric malabsorption leads to decreased absorption of intraluminal protein and fatty acids. Diet calcium binds to the increased amounts of intraluminal fatty acids instead of oxalate. Soluble free oxalate is now available for intestinal absorption leading to hyperoxalemia and hyperoxaluria.

The malabsorptive effect in certain bariatric procedures causing weight loss has often been discussed as the reason for enteric hyperoxaluria after MBS. Asplin^28^ explains this phenomenon as the result of fat malabsorption in the small intestine. Normally, intraluminal diet calcium binds to oxalate, builds an insoluble precipitate and is excreted in the feces. In postsurgical patients with fat malabsorption, diet calcium binds to the increased amounts of intraluminal fatty acids instead of oxalate. The soluble free oxalate reaches the colon and is available for passive and paracellular intestinal absorption (Fig. 4). Furthermore, intraluminal bile salts and fatty acids can also increase the membrane permeability in the bowel and thus augment oxalate absorption^29^. Various studies reported that especially restrictive types of bariatric surgery (e.g. sleeve gastrectomy, gastric banding) were not associated with an increased risk for postoperative hyperoxaluria or kidney stones^30–32^. In a study by Moreland et al. from 2017^33^, hyperoxaluria after RYGB correlated with the degree of steatorrhea, which was not the case before surgery. This supports the notion that MBS-associated hyperoxaluria derives from intestinal fat malabsorption.

It has also been proposed, that the risk for hyperoxaluria and CaOx-nephrolithiasis increases with the degree of malabsorption in bariatric procedures. The first study to report hyperoxaluria after RYGB in 2005^18^, distinguished between ‘standard RYGB’ (procedure in our study population) and ‘malabsorptive RYGB’. For the latter, length of the common channel was at 75–125 cm. Patients undergoing ‘malabsorptive RYGB’ showed a higher risk for hyperoxaluria and CaOx-nephrolithiasis. Lieske et al. corroborated those findings in 2015^20^, indicating that the degree of hyperoxaluria depends on the length of the remaining common channel and thus the amount of mucosa available for absorption.

In our study, patients with elevated oxalate excretion showed significantly greater total weight loss than controls. A significant positive correlation between weight loss and follow up time in hyperoxaluric patients supports this finding, whilst controls presented a negative correlation. The greater weight loss in hyperoxaluric patients could be explained through a more effective post-surgical malabsorption. To evaluate the state of malabsorption between the two groups, we determined serum levels of ‘malabsorption parameters’ (total protein, albumin, zinc and iron). We noticed significantly lower levels of serum protein in hyperoxaluric patients versus controls. This finding further points to a higher degree of malabsorption in hyperoxaluric patients.

EWL is a metric often used to determine the efficiency of bariatric surgery. To calculate EWL, three variables are necessary: pre- and postoperative weight and the patients’ ideal body weight (IBW)^34^. Unlike total weight loss, however, mean EWL showed no significant differences between hyperoxaluric patients and controls. This indicates, that delta weight loss, independent from reaching the individuals IBW, is a risk factor for developing hyperoxaluria after bariatric surgery.

### Limitations

This study has some limitations: first, the cohort was of moderate size due to the single center character limiting generalizability. Second, for determining the prevalence of hyperoxaluria in our cohort, a one-time 24 h urine collection was used to represent the patient´s urinary oxalate excretion. Hence, we cannot fully exclude false positives and false negatives. Furthermore, there was no report of pre-procedural urinary oxalate values allowing for exclusion of pre-existing hyperoxaluria as opposed to MBS-associated hyperoxaluria. Finally, the use of creatinine-based eGFR in the setting of bariatric surgery shall be interpreted with caution due to the post-surgical decrease of muscle and fat mass^22^.

## Conclusion

Our study demonstrates that hyperoxaluria is a common adverse event of malabsorptive bariatric surgery. We did not identify associated genetic determinants, rather the degree of postsurgical weight loss and levels of malabsorption serum parameters may allow for estimating the risk of enteric hyperoxaluria and consecutive kidney stone formation in the future.

## Supplementary Information

Supplementary Information.

## Data Availability

The authors declare that the data supporting the findings of this study are available within the article and its Supplementary information file. The raw data generated in this study are provided in the Supplementary Information/Source Data file. Source data are provided in this paper. Identified genetic variants were deposited in ClinVar (Submission ID: SUB13342722; Organization ID: 506086).

## Abbreviations

ACMG: American College of Medical genetics and Genomics
BMI: Body mass index
CADD: Combined annotation dependent depletion
CaOx-NL: Calcium-oxalate nephrolithiasis
CKD: Chronic kidney disease
ESKD: End-stage kidney disease
EWL: Excess weight loss
gnomAD: Genome Aggregation Database
HO: Hyperoxaluria
IBW: Ideal body weight
LOBB: Leipzig obesity biobank
MBS: Malabsorptive bariatric surgery
NAFLD: Non-alcoholic fatty liver disease
PH: Primary hyperoxaluria
RYGB: Roux-en-Y gastric bypass
tNGS: Targeted next generation sequencing
