# Supplementary material for: Delta weight loss unlike genetic variation associates with hyperoxaluria after malabsorptive bariatric surgery
Source: Sci Rep. 2023 Jun 3;13:9029. doi: 10.1038/s41598-023-35941-8 (PMC10239517; doi:10.1038/s41598-023-35941-8)
Supplement: Supplementary file 1 — Supplementary Information. [file 41598_2023_35941_MOESM1_ESM.docx]

**SUPPLEMENTARY FILES**

|  | ID | Sex | Age | Urinary oxalate (mmol/day) | Gene | Chromosome | Location | Nucleotide change | Amino acid change | Zygozity | gnomAD allele frequency NFE | CADD_Phred | clinvar ID | clinvar interpretation |
| --- | --- | --- | --- | --- | --- | --- | --- | --- | --- | --- | --- | --- | --- | --- |
| Hyperoxaluria | HOHO08 | F | 53 | 0.82 | *SLC26A1* | 4 | E3 | c.535C>T | p.Arg179Cys | het | 0.00021 | 24.7 | n.a. | n.a. |
|  | HOHO32 | F | 69 | 0.45 | *SLC26A1* | 4 | E4 | c.1231C>T | p.Arg411Trp | het | 0.00009 | 31 | 1028745 | VUS |
|  | HOHO35 | F | 62 | 1.01 | *GRHPR* | 9 | E4 | c.296G>A | p.Arg99Gln | het | 0.00017 | 24.8 | 914015 | VUS |
|  | HOHO47 | F | 64 | 0.48 | *SLC26A1* | 4 | E4 | c.2023C>T | p.Gln675Ter | het | 0 | 39 | n.a. | n.a. |
|  | HOHO63 | F | 71 | 1.13 | *SLC26A1* | 4 | E4 | c.1231C>T | p.Arg411Trp | het | 0.00009 | 31 | 1028745 | VUS |
|  | HOHO64 | F | 45 | 0.83 | *SLC26A1* | 4 | E4 | c.1231C>T | p.Arg411Trp | het | 0.00009 | 31 | 1028745 | VUS |
| Control | KOHO27 | F | 51 | 0.27 | *SLC26A1* | 4 | E4 | c.1231C>T | p.Arg411Trp | het | 0.00009 | 31 | 1028745 | VUS |
|  | KOHO29 | F | 52 | 0.18 | *SLC26A1* | 4 | E4 | c.1115G>T | p.Arg372Leu | het | 0.00007 | 21.6 | n.a. | n.a. |
|  |  |  |  |  | *SLC26A6* | 3 | E9 | c.1693-3_1698delCAGTGTGG | NA | hom | 0 | 25.5 | n.a. | n.a. |
|  | KOHO40 | M | 44 | 0.17 | *SLC26A7* | 8 | E7 | c.809C>A | p.Ser270Ter | het | 0.00002 | 44 | n.a. | n.a. |
|  | KOHO42 | F | 48 | 0.44 | *SLC26A1* | 4 | E4 | c.1970T>C | p.Leu657Pro | het | 0 | 22.9 | n.a. | n.a. |
|  | KOHO49 | F | 48 | 0.37 | *AGXT* | 2 | E8 | c.87_134delinsGTCTCACCCATGTTCCCACCCACA | p.Pro30_Met45delinsSerHisProCysSerHisProGln | het | 0 | 24.8 | n.a. | n.a. |
|  | KOHO76 | M | 54 | 0.32 | *SLC26A1* | 4 | E4 | c.1231C>T | p.Arg411Trp | het | 0.00009 | 31 | 1028745 | VUS |

**Table S1: Variant table.** Abbreviations: F, female; het, heterozygous; hom, homozygous; M, male; NFE, Non-Finnish European; VUS, variant of uncertain significance

**Table S2: Secondary diseases.** Abbreviations: DM, diabetes mellitus; NAFLD, non-alcoholic fatty liver disease

| **Parameters** | **With diagnosis** | **Without diagnosis** | **Total (n=67)** | ***p*-value** |
| --- | --- | --- | --- | --- |
| **Hypertension** |  |  |  |  |
| **HO, n (%)** | n=21/29 (72%) | n=8/29 (28%) |  | 0.78 |
| **Control, n (%)** | n=29/38 (76%) | n=9/38 (24%) |  |  |
| **Oxalate/24h [mmol/d]** | 0.45 ± 0.03 (n=50) | 0.50 ± 0.07 (n=17) | 0.46 ± 0.03 (n=67) | 0.51 |
| **DM Type 2** |  |  |  |  |
| **HO, n (%)** | n=9/29 (31%) | n=20/29 (69%) |  | 0.79 |
| **Control, n (%)** | n=10/38 (26%) | n=28/38 (74%) |  |  |
| **Oxalate/24h [mmol/d]** | 0.47 ± 0.04 (n=19) | 0.46 ± 0.04 (n=48) | 0.46 ± 0.03 (n=67) | 0.87 |
| **NAFLD** |  |  |  |  |
| **HO, n (%)** | n=11/29 (38%) | n=18/29 (62%) |  | 0.80 |
| **Control, n (%)** | n=13/38 (34%) | n=25/38 (66%) |  |  |
| **Oxalate/24h [mmol/d]** | 0.45 ± 0.04 (n=24) | 0.47 ± 0.04 (n=43) | 0.46 ± 0.03 (n=67) | 0.80 |


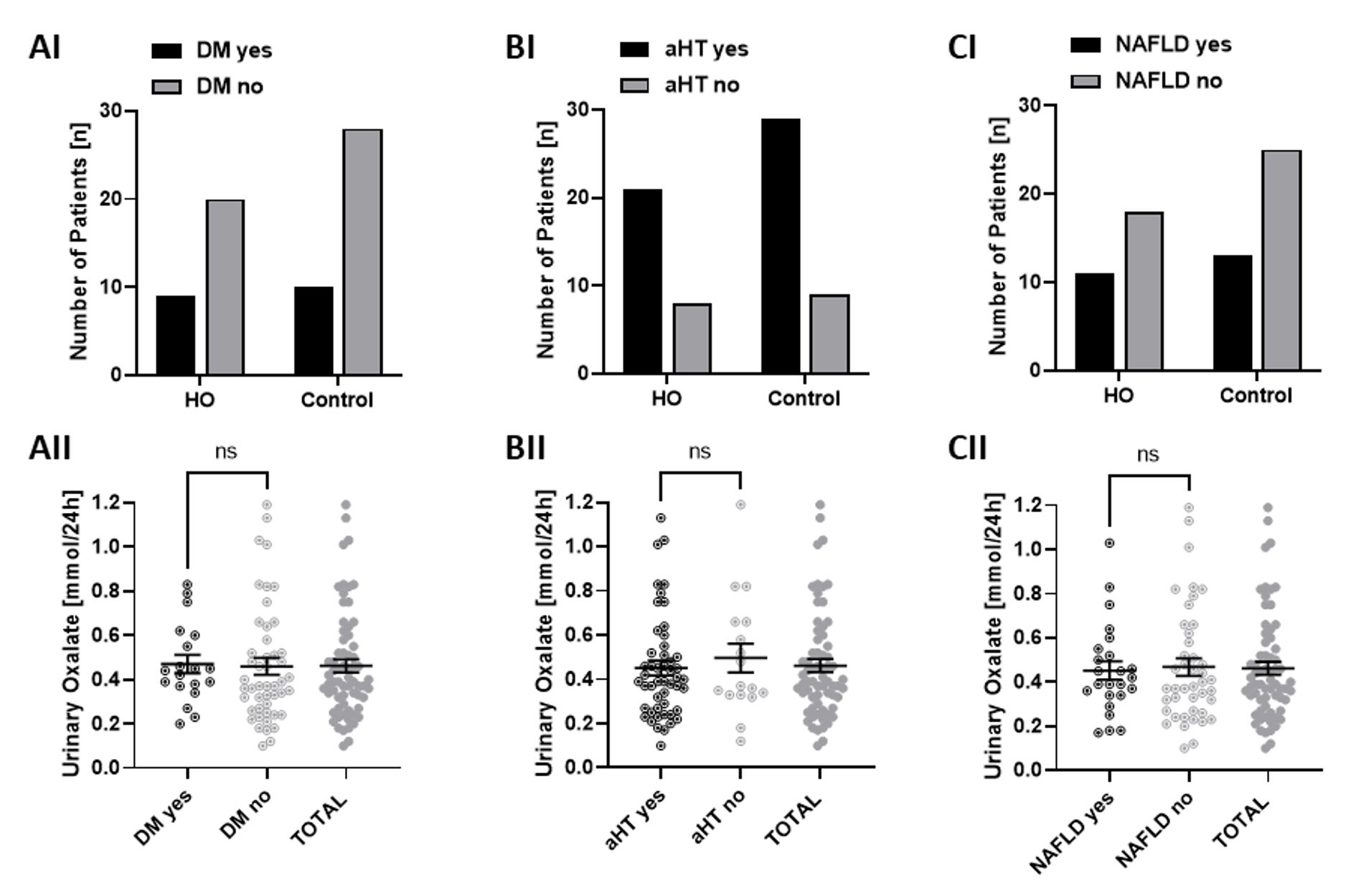


**Figure S1: Association of hyperoxaluria and secondary diagnoses.** (**AI – CI**) Bar graph comparing the frequency distribution of secondary diseases (DM, aHT, NAFLD) between HO and control. Fisher’s exact test was used for statistical analysis. (**AII – CII**) Values of urinary oxalate excretion in patients with or without secondary diseases expressed as mean ± SEM. aHT, arterial Hypertension; DM, diabetes mellitus; HO, hyperoxaluria; NAFLD, non-alcoholic fatty liver disease; ns, not significant
